# Age Dependent Partitioning Patterns of Essential Nutrients Induced by Copper Feeding Status in Leaves and Stems of Poplar

**DOI:** 10.3389/fpls.2022.930344

**Published:** 2022-07-05

**Authors:** Cameron Hunter, Jared J. Stewart, Sean M. Gleason, Marinus Pilon

**Affiliations:** ^1^Department of Biology, Colorado State University, Fort Collins, CO, United States; ^2^Water Management and Systems Research Unit, Agricultural Research Service, United States Department of Agriculture, Fort Collins, CO, United States; ^3^Department of Ecology and Evolutionary Biology, University of Colorado, Boulder, Boulder, CO, United States

**Keywords:** nutrition, copper deficiency, macronutrients, micronutrients, homeostasis, metabolism, partitioning

## Abstract

Copper (Cu) is an essential micronutrient, and its deficiency can cause plants to undergo metabolic changes at several levels of organization. It has been shown that leaf age can play a role in nutrient partitioning along the shoot axis of poplar. In this study, we investigated the effect of Cu deficiency on the altered partitioning of essential macro and micronutrients in leaves and stems of different age. Cu deficiency was associated with higher concentrations of calcium, magnesium, sulfur, iron, zinc, manganese, and molybdenum in leaves and relatively higher concentrations of calcium, phosphorus, iron, and zinc in stems. Leaf and stem age had significant effects on nutrient partitioning. Principal component analyses revealed patterns that point to inverse influences in leaves and stems on nutrient partitioning. Specifically, these analyses revealed that nutrient partitioning in leaves was influenced by Cu feeding status more than developmental stage, whereas nutrient partitioning in stems was influenced by developmental stage more than Cu feeding status. These results suggest that Cu deficiency and developmental stage can significantly influence the partitioning and homeostasis of macro and micronutrients in poplar organs.

## Introduction

Mineral nutrients are needed in varying amounts by plant species and can be classified as macro- or micronutrients. Macronutrients—including nitrogen (N), phosphorus (P), potassium (K), calcium (Ca), magnesium (Mg), and sulfur (S)—are needed at 1,000 μg g^–1^ DW (i.e., ppm) or higher, while micronutrients—including copper (Cu), iron (Fe), zinc (Zn), manganese (Mn), boron (B), molybdenum (Mo), and nickel (Ni)—are needed at 0.1–100 μg g^–1^ DW (Epstein and Bloom, 2005; Marschner, 2012). Macro- and micronutrients are utilized in a wide variety of structural and physiological plant processes (Hänsch and Mendel, 2009; Maathuis, 2009), and the metabolism of several of these essential nutrients are tightly linked. For example, Cu toxicity has been shown to affect S metabolism in Chinese cabbage resulting in altered uptake and distribution of sulfate (Shahbaz et al., 2010). Developmental stage can also contribute to macro- and micronutrient distribution. In hybrid poplar, we recently observed that pulse experiments with ^65^Cu resulted in the favored partitioning to young leaves and young stems after a period of Cu deficiency (Hunter et al., 2022).

Soil or media that lack essential micronutrients can cause nutrient-specific deficiency symptoms in plant tissues and trigger metabolic remodeling at several levels of organization (Marschner, 2012; Billard et al., 2014; Shahbaz et al., 2015). For instance, when Cu becomes deficient, a response is triggered at the molecular level which is referred to as the Cu economy system (Ravet et al., 2011; Kropat et al., 2015; Shahbaz et al., 2015). Essentially, once Cu limitation is sensed, targets of the transcription factor SPL7 (SQUAMOSA promoter-binding protein-like) are upregulated (Bernal et al., 2012). SPL7 then upregulates the expression of several microRNAs (Cu-microRNAs; Pilon, 2017) that serve to mediate the degradation of mRNAs encoding for target Cu proteins that apparently are indispensable (Yamasaki et al., 2009; Bernal et al., 2012). The available Cu pool is hypothesized to be utilized in essential Cu proteins including plastocyanin to continue photosynthetic electron transport (Ravet et al., 2011; Shahbaz et al., 2015). While metabolic remodeling after Cu deficiency has been shown to occur at the molecular level by affecting gene expression, Cu nutrient deficiency can also affect allocation of other essential nutrients in plant organs. This demand can vary greatly along the axis of the shoot and differ depending on the nutrient, organ, and developmental stage under consideration (Siebrecht et al., 2003). Recently, Assunção et al. (2022) argued that a better understanding of nutrient interactions and partitioning within the plant should facilitate efforts to improve agricultural sustainability as well as nutritional quality of agricultural products, especially in areas with soil micronutrient deficiencies.

Given the different demand for elements of various plant organs, we were interested to investigate how Cu deficiency affects nutrient partitioning in different plant parts. Because of the possibility of crosstalk between homeostasis of micronutrients (Pilon et al., 2009; Rai et al., 2021), we hypothesized that Cu deficiency will alter the nutrient partitioning especially for Mo, Mn, Fe, and Zn. The objective for this study was to examine how Cu deficiency altered the partitioning of mineral macronutrients (Ca, Mg, P, K, and S) and micronutrients (Fe, Zn, Mn, and Mo) in leaves and stems of different age in hybrid poplar.

## Materials and Methods

### Plant Material and Growth Conditions

Hybrid white poplar (*Populus tremula* × *P. alba*, INRA 717-1B4) were grown hydroponically as described by Hunter et al. (2022). Seedlings were propagated *in vitro* on ½ strength Murashige and Skoog (Sigma-Aldrich, St. Louis, MO, United States) hormone free media with sucrose (20 g L^–1^) and agar (7 g L^–1^). Explants that were rooted and approximately 8 cm tall with an average age of 3 months were randomly distributed to 20-L black plastic buckets (3 plants per bucket) with an aerated one-tenth strength modified Hoagland’s solution (Hoagland and Arnon, 1950). The hydroponic solution pH was adjusted to 5.9 with KOH. The hydroponic solution for the Cu-sufficient treatment (+Cu) included 50 nM CuSO_4_. The hydroponic solution for the Cu-deficient treatment (–Cu) was the same as that for +Cu except CuSO_4_ was omitted. Plants were then grown for 6 weeks in a climate-controlled room under a 16 h photoperiod of 150 μmol photons m^–2^ s^–1^ (and 8 h dark period) with air temperature maintained at 22 ± 1°C.

### Chlorophyll Fluorescence

Characterized leaves from each plant were excised between 6 and 8 h in the photoperiod, the petioles were placed in DI water, and the leaves were darkened for 15 min before measurements of minimal (*F*_o_) and maximal (*F*_m_) chlorophyll fluorescence with an FMS system (Hansatech, Norfolk, United Kingdom) or a MAXI version IMAGING-PAM (Heinz Walz GmbH, Effeltrich, Germany). Steady-state (*F*), maximal (*F*_m_′), and minimal (*F*_o_′) chlorophyll fluorescence were then measured under a series of increasing light intensities as described previously (Cohu and Pilon, 2007; Hunter et al., 2022). Photosystem II (PSII) efficiency was calculated as (*F*_m_ – *F*_o_)/*F*_m_ = *F*_v_/*F*_m_ (maximal efficiency in darkness) or (*F*_m_′ – *F*)/*F*_m_′ (operating efficiency in light; Genty et al., 1989), and PSII reduction state (i.e., the proportion of PSII reaction centers that are closed) was calculated as (*F* – *F*_o_′)/(*F*_m_′ – *F*_o_′) = 1 – *q*_*P*_ (for details, see Demmig-Adams et al., 1996).

### Elemental Analysis

Concentrations of the macronutrients Ca, K, Mg, P, and S and the micronutrients Fe, Mo, Mn, and Zn were determined in the youngest leaf tissue (Leaf 0–2) as well as the five antecedent leaves (Leaf 3, Leaf 4, Leaf 5, Leaf 6, and Leaf 7; for representative images, see Supplementary Figure 1). In our –Cu treatment, Leaf 7 was approximately the first leaf to develop fully out of tissue culture. Additionally, concentrations of these macro- and micronutrients were determined in relatively young and mature sections of stem. Young stems were parts of stems above Leaf 7, and mature stems were parts of stems below Leaf 7. Collected leaves and stem sections were oven dried at 55°C for 72 h. Approximately 100 mg of plant material was digested in 1 mL of HNO_3_ (70%). The digest was heated for 2 h at 60°C and 6 h at 130°C and subsequently diluted up to 10 mL with double distilled water. Samples were analyzed using an ELAN-DRC Inductively Coupled Plasma-Optical Emission Spectroscopy instrument (Pilon-Smits et al., 1999).

### Statistical Analysis

Statistical analyses were performed using R 4.0.3^1^ and JMP Pro 15.0.0 (SAS Institute Inc., Cary, NC, United States) software. One-way ANOVAs were performed using the “lm” function and Tukey mean separation was done using the “emmeans” package (Lenth et al., 2022) at a significance level of 0.05. Two-sample *t*-tests were performed with the “t.test” function with a significance level of 0.05. Two-way ANOVAs and principal component analysis were performed using JMP Pro 15.0.0. Data visualization were done using the “ggplot2” package (Wickham, 2016).

## Results

### Poplar Growth and Photosynthetic Performance

**FIGURE 1:**
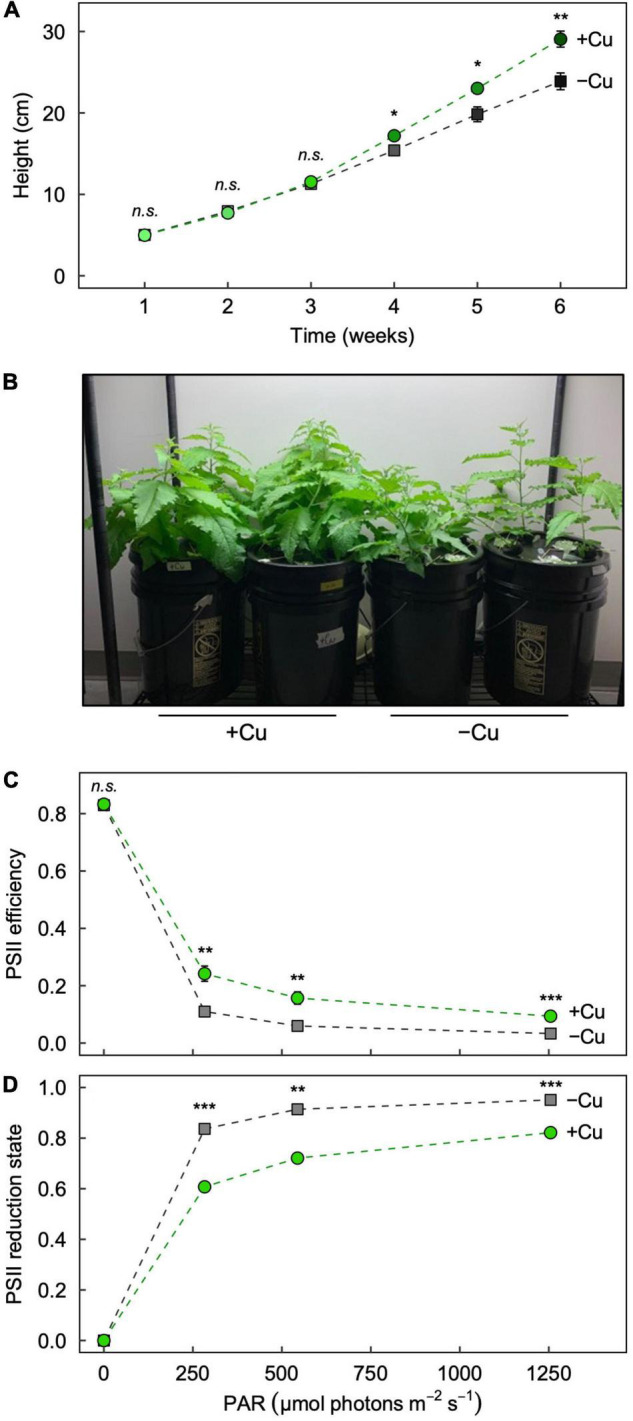
Effect of Cu deficiency on plant growth and photosynthesis. **(A)** Plant height over the 6-week growth period, **(B)** images of plants at the end of the 6-week growth period, and the response of **(C)** photosystem II (PSII) efficiency and **(D)** PSII reduction state to increasing light intensity in Leaf 3 of hybrid white poplar (*P. tremula* × *P. alba*) grown in +Cu (green circles) and –Cu (gray squares) conditions. Mean values ± SE (*n* = 6). The asterisks denote significant differences; *P < 0.05, **P < 0.01, ***P < 0.001 (*n.s.*, not significantly different).

We first aimed to confirm that our –Cu treatment produced expected phenotypic symptoms of Cu deficiency in hybrid white poplar (*P. tremula* × *P. alba*) after 6 weeks of growth. The plants grew similarly until week 4 (Figure 1A), the time at which Cu deficiency symptoms (stunted growth, leaf curling, and chlorosis) typically appear in our hydroponic system (e.g., Hunter et al., 2022). Plants in +Cu conditions outgrew those in –Cu conditions through week 6 (Figure 1B), with significant differences in weeks 4, 5, and 6 (Figure 1A).

Cu deficiency lowers the efficiency of photosynthetic electron transport and causes the plastoquinone pool to be more reduced (e.g., Shahbaz et al., 2015; Hunter et al., 2022), and these effects can be assessed *via* nonintrusive measurements of chlorophyll fluorescence (Demmig-Adams et al., 1996). As previously reported (Hunter et al., 2022), the maximal efficiency of photosynthetic electron transport in darkness (*F*_v_/*F*_m_) was similarly high in leaves from +Cu and –Cu conditions (Figure 1C), but the operating PSII efficiency when exposed to increasing light intensities was significantly and consistently greater in Leaf 3 from +Cu versus –Cu conditions (Figure 1C). Likewise, PSII reduction state (i.e., the proportion of closed PSII reaction centers) was significantly greater in Leaf 3 from –Cu versus +Cu conditions under all measured light intensities (Figure 1D). Generally, operating PSII efficiency was higher in mature (Leaf 7) versus young (Leaf 3) leaves in both +Cu and –Cu conditions (Supplementary Figure 2).

### Copper Deficiency and Mineral Partitioning as a Function of Age

**FIGURE 2:**
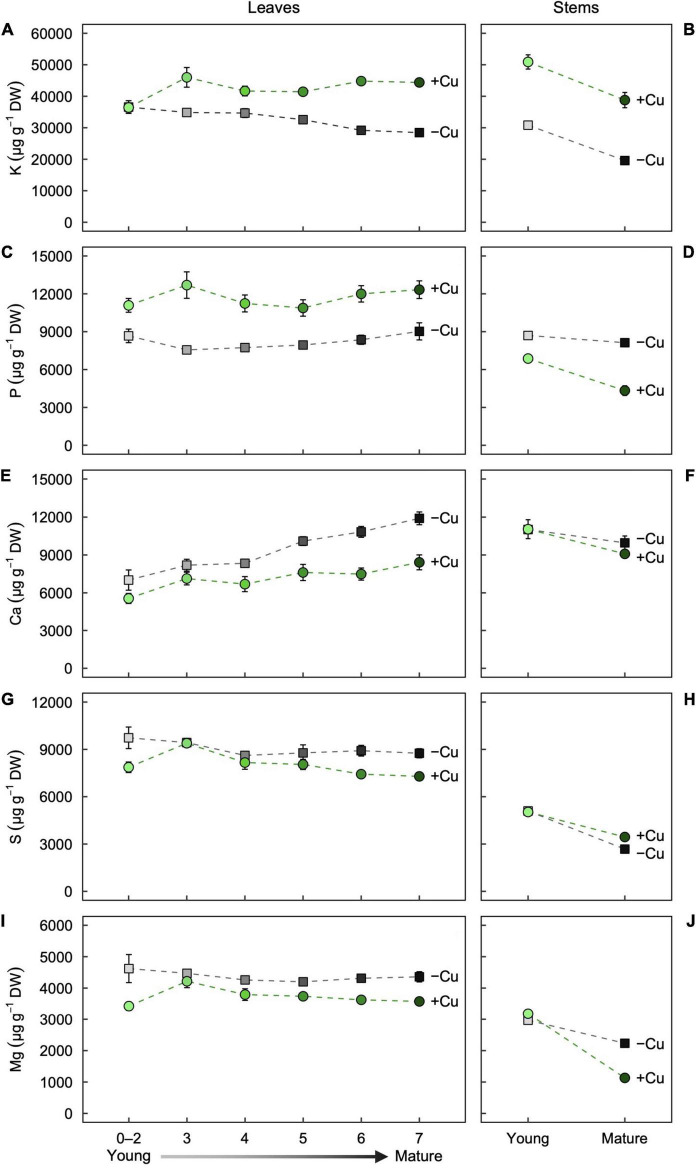
Partitioning of the macronutrients **(A,B)** K, **(C,D)** P, **(E,F)** Ca, **(G,H)** S, and **(I,J)** Mg as a function of age in **(A,C,E,G,I)** leaves and **(B,D,F,H,J)** stems of hybrid white poplar (*P. tremula* × *P. alba*) grown in +Cu (green circles) and –Cu (gray squares) conditions. Mean values ± SE (*n* = 6). Results of two-way ANOVAs for the effects and interaction of tissue age and Cu feeding status on mineral partitioning in leaves as well as stems are shown in Table 1.

**FIGURE 3:**
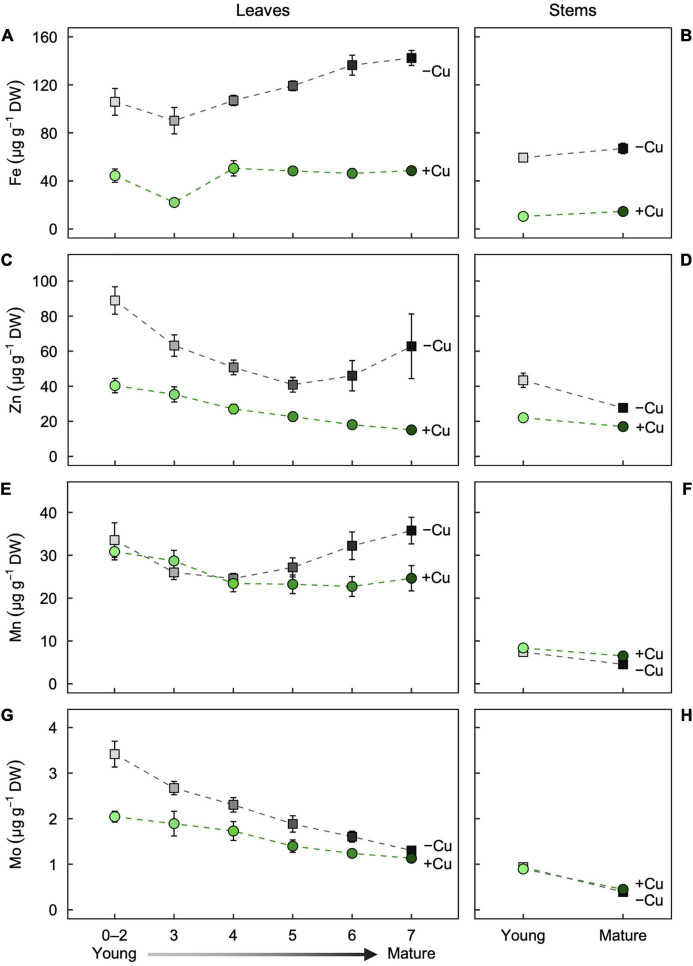
Partitioning of the micronutrients **(A,B)** Fe, **(C,D)** Zn, **(E,F)** Mn, and **(G,H)** Mo as a function of age in **(A,C,E,G)** leaves and **(B,D,F,H)** stems of hybrid white poplar (*P. tremula* × *P. alba*) grown in +Cu (green circles) and –Cu (gray squares) conditions. Mean values ± SE (*n* = 6). Results of two-way ANOVAs for the effects and interaction of tissue age and Cu feeding status on mineral partitioning in leaves as well as stems are shown in Table 1.

We next investigated how Cu deficiency alters the mineral nutrition of leaves and stems of different age. We defined a developmental gradient of leaf age along the shoot of hybrid poplar by measuring the first seven leaves of +Cu and –Cu plants (Supplementary Figure 1). Young stems were the stem portion from the apical meristem to Leaf 7 and mature stem was below Leaf 7. Overall, Cu deficiency altered mineral nutrient concentrations in all measured organ types (Figures 2, 3). A brief description of the results for each element is given below.

#### Partitioning of Macronutrients

In the youngest leaves (Leaf 0–2), Potassium (K) concentration were almost identical between the treatments (Figure 2A). K concentration in Leaf 3–7 was slightly variable in +Cu plants (Figure 2A). In –Cu leaves, K concentration decreased steadily with increasing leaf maturity although the effect of leaf age on K concentration was non-significant (Figure 2A and Table 1). Stems showed similar responses with K concentration present a lower concentration in –Cu conditions (Figure 2B). Young stems showed a 60%, and old stems showed 50% reduction in K concentration, respectively, (Figure 2B). Cu treatment and stem age had independent significant effects on K partitioning (Table 1).

**TABLE 1:** Results of two-way ANOVAs for the effects and interaction of tissue age and Cu feeding status on mineral partitioning in leaves as well as stems of hybrid white poplar (*P. tremula* × *P. alba*) grown in +Cu and –Cu conditions.

Mineral(s)	Leaves			Stems		
	Age	Cu	Age × Cu	Age	Cu	Age × Cu
Ca	***	***	*n.s.*	*	*n.s.*	*n.s.*
Mg	*n.s.*	***	*n.s.*	***	**	***
P	*n.s.*	***	*n.s.*	***	**	***
K	*n.s.*	***	***	***	***	*n.s.*
S	**	***	*n.s.*	***	*	*
Fe	***	***	*	*n.s.*	***	*n.s.*
Zn	**	**	*n.s.*	**	***	*
Mn	*	***	*n.s.*	**	*	*n.s.*
Mo	***	***	*	***	*n.s.*	*n.s.*
PC1_Leaves_/PC1_Stems_	*n.s.*	***	*n.s.*	***	*n.s.*	*n.s.*
PC2_Leaves_/PC2_Stems_	***	***	***	*	***	*

The asterisks denote significant effects; *P < 0.05, **P < 0.01, ***P < 0.001 (n.s. = not significant).

P was variable across leaf age in +Cu plants with Leaf 3 having the highest concentration (Figure 2C). Cu deficiency seemed to alter P concentration across leaf age with –Cu plants possessing lower concentration compared to +Cu plants, although leaf age did not have a significant effect on P concentration (Figure 2C and Table 1). In –Cu stems, there was an 47% increase in P concentration for mature stems and a 21% increase in young stems (Figure 2D). Cu treatment and stem age both had an independent significant effect on P concentration in stems (Table 1).

Ca was distributed to Leaf 5–7 at a higher concentration versus Leaf 0–2, 3, and 4 in Cu sufficient plants (Figure 2E). The same trend was observed in –Cu leaves (Figure 2E). Ca concentrations were higher in –Cu leaves compared to +Cu leaves (Figure 2E). Cu and leaf age had independent significant effects on Ca partitioning, but the Cu by age interaction was not significant (Table 1). Ca concentration in young stems were almost identical between +Cu and –Cu treatments with older stems containing less Ca (Figure 2F). Stem age had a small but significant effect on Ca partitioning in young and old stem (*P* = 0.009; Figure 2F).

S concentration were 79% lower in –Cu Leaf 0–2 than +Cu Leaf 0–2 (Figure 2G). Leaf 3 had the highest S concentration in both treatments (Figure 2G). We observed a steady decrease from Leaf 4 to Leaf 7 in S concentration (Figure 2G). Leaf age had a significant effect (*P* = 0.005) on S partitioning in leaves, but Cu treatment did not (Table 1). S concentration in young stems were almost identical between treatments (Figure 2H). There was 22% less S in –Cu mature stems compared to +Cu mature stem (Figure 2H). There were significant effects on S partitioning from stem age and Cu treatment as well as a significant age by Cu interaction (Table 1).

Mg concentrations were relatively uniform across leaf age in +Cu leaves with Leaf 3 having a slightly higher concentration (Figure 2I). A similar trend was observed for Mg in –Cu leaves, but Mg concentration were higher in all –Cu leaves (Figure 2I). Cu treatment was the only factor with a significant effect on Mg partitioning (Table 1). Stem age had a highly significant effect in +Cu (Table 1), with Mg concentration were approximately 2,000 μg g^–1^ DW greater in young stems versus mature stems (Figure 2J and Table 1). Mg in Cu deficient mature stems was present at very low concentrations (186.7 μg g^–1^ DW) and 84% lower than +Cu Mg concentration in mature stem (Figure 2J).

#### Partitioning of Micronutrients

Fe in –Cu plants were higher in all leaves compared to +Cu plants. Leaf age also had a highly significant effect on Fe partitioning (Figure 3A). In Cu deficient Leaf 0–2, Fe was 60% higher and 76% higher in Leaf 3 versus +Cu leaves (Figure 3A). Fe concentration in –Cu plants were 60% higher in Leaves 4–7 compared to +Cu Leaves 4–7 (Figure 3A). The same observation was made for young and mature stems (Figure 3B). Fe concentration in Cu deficient stems were approximately three-fold higher than +Cu stems with Cu treatment having the only significant effect (Figure 3B and Table 1).

Zn concentrations in leaves first decreased with age (Leaf 0–5) and then increased with age (Leaf 6–7) in the –Cu treatment (Figure 3C), whereas the leaf Zn concentrations in the +Cu treatment steadily decreased with age (Figure 3C). Leaf age and Cu treatment had independent significant effects on Zn concentration, but not a significant interaction (Table 1). Zn was higher for both young leaves and mature stems in the –Cu treatment (Figures 3C,D). Age and Cu treatments had significant but slightly contrasting effects on Zn partitioning (Figure 3D and Table 1).

Mn concentration was similar in Leaf 4–7 of +Cu plants and present at higher concentration in Leaf 0–2 and Leaf 3 (Figure 3E). In –Cu plants, Mn was lower in Leaf 3 and Leaf 4 while higher in all other leaves (Figure 3E). Cu deficient leaves had higher Mn concentration compared to +Cu leaves in Leaf 0–2 and Leaf 4–7 with leaf age have a significant effect on Mn concentration (Figure 3E and Table 1). Mn was higher in +Cu stems than –Cu stems with an individual but not combined significant effect on Mn partitioning (Figure 3F and Table 1).

**FIGURE 4:**
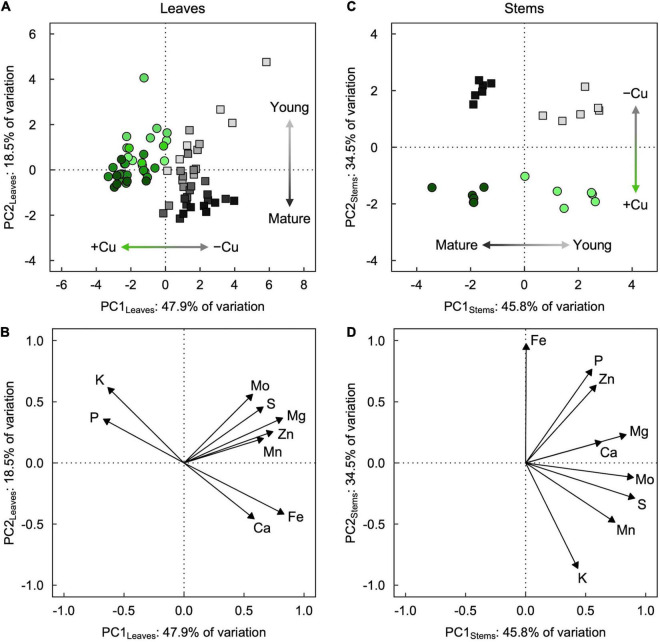
**(A,C)** Score and **(B,D)** loading plots of the first two principal components from principal component analyses (PCA) of mineral partitioning in **(A,B)** leaves and **(C,D)** stems of hybrid white poplar (*P. tremula* × *P. alba*) grown in +Cu (green circles) and –Cu (gray squares) conditions. Results of two-way ANOVAs for the effects and interaction of tissue age and Cu feeding status on mineral partitioning in leaves as well as stems are shown in Table 1.

Mo had a similar trend in leaf age as Zn with older leaves having less Mo in both +Cu and –Cu treatments (Figures 3C, 4G). Leaf 7 (oldest) was the only leaf age that had higher Mo in the –Cu treatment but only by 13% (Figure 3G). Mo was the only element besides Fe to have significant individual treatment effects (age, Cu) as well as a significant age by Cu interaction effect on element partitioning (Table 1). Mature stem concentration on Mn were similar between +Cu and –Cu conditions (Figure 3H). Cu deficient young stems had 85% more Mo compared to +Cu young stems (Figure 3H). The only significant effect found in stems was stem age (Table 1).

### Evaluation of Mineral Composition Across Leaves and Stems

Principal component analysis revealed slightly contrasting influences of tissue age and Cu treatment on overall mineral partitioning in leaves versus stems (Figure 4). For leaves (Figures 4A,B), the first two principal components, PC1_Leaves_ and PC2_Leaves_, explained approximately two-thirds (47.9% and 18.5%, respectively) of the variation in the macro- and micronutrient concentrations. Leaves from +Cu and –Cu conditions (regardless of age; Table 1) separated almost entirely along PC1_Leaves_ (Figure 4A), with mostly negative PC1_Leaves_ scores for +Cu conditions and mostly positive PC1_Leaves_ scores for –Cu conditions. Leaves of different age separated along PC2_Leaves_ (Table 1), with relatively lower PC2_Leaves_ scores for older leaves and higher PC2_Leaves_ scores for younger leaves (Figure 4A). The separation by leaf age along PC2_Leaves_ was more exaggerated in the –Cu conditions compared to the +Cu conditions (Table 1), and PC2_Leaves_ scores were generally higher in +Cu versus –Cu conditions (Table 1). The minerals underlying these trends formed two distinct clusters: (i) Mg, S, Zn, Mo, and Mn, and (ii) Fe, Ca, K, and P (Figure 4B). The second cluster consisted of two inversely associated pairings that coincided with the separation of Cu sufficiency (higher K and P) and Cu deficiency (higher Fe and Ca).

For stems (Figures 4C,D), the first two principal components, PC1_Stems_ and PC2_Stems_, explained over 80% (45.8 and 34.5%, respectively) of the variation in the macro- and micronutrient concentrations. Stems of different ages (regardless of Cu conditions; Table 1) separated entirely along PC1_Stems_ (Figure 4C), with negative PC1_Stems_ scores for older stems and positive PC1_Stems_ scores for young stems. Stems from +Cu and –Cu conditions separated entirely along PC2_Stems_ (Figure 4C), with negative PC2_Stems_ scores for +Cu conditions (similar for mature and young stems) and positive PC2_Stems_ scores for –Cu conditions (slightly higher in mature versus young stems; Table 1). PC1_Stems_ was loaded predominantly (79.7%) by S, Mo, Mg, Mn, and Ca, whereas PC2_Stems_ was loaded predominantly (86.6%) by Fe, P, Zn, and K (Figure 4D). The latter cluster of minerals consisted of two inversely associated subgroups that coincided with the separation of +Cu conditions (higher K) and –Cu conditions (higher Fe, P, and Zn).

## Discussion

Cu deficiency resulted in higher concentrations of Ca, Mg, S, Fe, Zn, Mn, and Mo in leaves and higher concentrations of Ca, P, Fe, and Zn in stems. Additionally, there were significant effects of leaf and stem age on mineral partitioning for all measured elements suggesting that age is also an important determinant of mineral partitioning.

Interestingly, for several elements, the change in concentration due to Cu deficiency was highly dependent on tissue type. For instance, P concentration were higher in all leaves of +Cu plants and lower in –Cu plants, but higher in Cu deficient young and mature stems, respectively. In leaves, Cu deficiency caused increases in all micronutrient concentration except for Mn concentration in Leaf 3. The largest quantitative differences between +Cu and –Cu treatments for micronutrients can be seen in Fe and Zn concentration in leaves and stems. This difference could be due to the cross talk between Fe and Cu homeostasis (Carrió-Seguí et al., 2019; Rai et al., 2021), such as increased activities of enzymes with a Fe cofactor (e.g., Fe superoxide dismutase) that could compensate for decreased activities of functionally analogous enzymes that require a Cu cofactor (e.g., Cu/Zn-superoxide dismutase; Kliebenstein et al., 1998; Cohu and Pilon, 2007). Perea-García et al. (2020) observed an increase in root and shoot Fe concentrations in wild type *Arabidopsis* under Cu deficiency, and Waters and Armbrust (2013) found increased Cu concentrations under Fe deficiency. Additionally, it has been observed that Cu deficiency induces the expression of Cu-II chelate reductase activity in root tips (Bernal et al., 2012). In *Arabidopsis*, these reductases are encoded by ferric reductase/oxidase 4/5, which are conserved in plants (Bernal et al., 2012).

In our data, Mo concentration decreased as leaves became older and were higher in the –Cu treatment. Crosstalk between Cu and Mo homeostasis is not unexpected, given that Cu is required for the synthesis of Molybdenum co-factor (Kuper et al., 2004). Mo also followed the same partitioning trend as S in leaves. Mo is taken up as molybdate and oxyanion is taken up by sulfate family member transporters (Tomatsu et al., 2007). Billard et al. (2014) studied the effects of Cu deficiency on Cu remobilization, Mo accumulation, and chloroplast protein changes in *Brassica napus*. It was observed that Cu deficiency had no effect on N, Ca, K, S, P, B, Fe, Mn, and Zn uptake (Billard et al., 2014). However, the authors did observe that Mo uptake increased by 121% and the increased expression of a Mo uptake gene (*MOT1*) occurred under Cu deficiency (Billard et al., 2014). We see a more broad effect of Cu deficiency in hybrid poplar where in most cases, leaf age and Cu status had a significant effect on mineral partitioning except for Mg, P, and K.

Siebrecht et al. (2003) examined the diurnal variations in nutrient concentrations in the xylem sap of hydroponically grown poplar. These investigators observed that Mg, Ca, K, NO_3_^–^, H_2_PO_4_^–^, and SO_4_^2–^ reached their maximum concentrations in the first half of a 16 h photoperiod while photosynthesis and transpiration remained constant (Siebrecht et al., 2003). This study also examined leaf age finding that K concentration remained constant from young leaves to old leaves (37 leaf plants), Mg and Ca concentration were higher in older leaves, and S plateaued at Leaf 10 of 37 (Siebrecht et al., 2003). Photosynthesis and transpiration rates were relatively low in the youngest leaves and higher in slightly older leaves (Siebrecht et al., 2003), which are consistent with the findings of the current study (see also Shahbaz et al., 2015) as well as those reported recently by Hunter et al. (2022), respectively. In the latter study, we reported that a similar age-dependent effect on stomatal conductance under Cu deficiency was reversed (i.e., stomatal conductance of the youngest leaves exceeded that of older leaves) shortly after Cu was resupplied (Hunter et al., 2022). It is likely that any spatiotemporal differences in leaf hydraulic and photosynthetic activities (as well as supporting anatomical features; see Lawrence et al., 2021) have important consequences on mineral composition across the plant due to their direct influence on xylem activity (Brodribb et al., 2007).

Remobilization of nutrients is another strategy used by plants when nutrient deficiency or leaf senescence occurs (Hill, 1980; Marschner, 2012), and the extent of remobilization varies across species (Maillard et al., 2015). Maillard et al. (2015) examined remobilization of 13 nutrients during nutrient deficiency and leaf senescence in cultivated crop and woody species. The authors found a low net remobilization efficiency for field grown black poplar (*Populus nigra*) for all measured nutrients compared to English oak (*Quercus robur*; Maillard et al., 2015). Under Cu deficiency, *B. napus* was found to have a low remobilization score (%) as well (Billard et al., 2014). It could be that the 3 weeks of Cu deficiency increases the onset of leaf senescence in some species (cf. Ishka and Vatamaniuk, 2020), which could also trigger early signals for nutrient remobilization (Billard et al., 2014). However, the remobilization of some nutrients is seemingly constitutively greater than others and depends, in part, on transport out of the leaves *via* the phloem (Hill, 1980; Marschner, 2012). For example, the concentration of nutrients with relatively low mobility, such as Ca (Biddulph et al., 1959), increased with leaf maturity in +Cu and –Cu conditions. These results are consistent with those reported by Maillard et al. (2015), who observed a constant accumulation of Ca over the lifetime of leaves in poplar (see also Siebrecht et al., 2003). Similarly, Maillard et al. (2015) reported Mo remobilization was relatively high in *P. nigra*, which is consistent with our findings that Mo concentrations decreased with leaf age.

Our data revealed significant interactions between Cu status, tissue type, and tissue age. We employed principal component analysis, which has been utilized effectively to synthesize and evaluate ionomic data in similar studies (e.g., Baxter et al., 2008). Our results show clear distinctions that suggest mineral partitioning in leaves is influenced heavily by Cu deficiency and slightly by leaf age. Surprisingly, Fe did not cluster with the same group as Mo, Mn, and Zn, even though all of these micronutrients contributed to the separation of leaves from +Cu and –Cu conditions. Instead, Fe clustered with Ca whose concentrations were higher in mature leaves, likely due to their relatively low mobility in poplar (Maillard et al., 2015). Furthermore, K and P did cluster together with the same trend in leaves (higher concentration in +Cu conditions). The way Mo clustered in the loadings plot could reflect how it was partitioned distinctly from Mn and Zn regarding mature leaves (e.g., Zn and Mn concentrations increased in the two oldest leaves measured). The separation of stems along PC1_Stems_ and PC2_Stems_ revealed a clustering that suggests developmental stage has a strong influence on mineral partitioning and, to a lesser degree, Cu status. It has been suggested before that Cu delivery is prioritized to leaves for use in photosynthesis after deficiency, but Cu prioritization has been less clear for stems (Shahbaz et al., 2015).

## Conclusion

In conclusion, Cu deficiency revealed quantitative changes in leaf and stem mineral concentration and possibly alteration in uptake strategies due to Cu deficiency. Specifically, Cu deficiency resulted in higher mineral concentrations of Ca, Mg, S, Fe, Zn, Mn, and Mo in leaves and higher mineral concentrations of Ca, P, Fe, and Zn in stems. Principal component analysis revealed a clear influence of organ age on mineral partitioning in leaves and stems. Principal component analysis also revealed distinct clusters of elements that responded similarly to Cu deficiency (Mn, Mg, S, Mo, and Zn).

## Data Availability Statement

The raw data supporting the conclusions of this article will be made available by the authors, without undue reservation.

## Author Contributions

CH and MP conceived and designed the study. CH carried out the experiments. CH, MP, JS, and SG analyzed the data. CH wrote the manuscript with input and contributions from MP, JS, and SG. All authors have read and agreed to the final version of the manuscript.

## Conflict of Interest

The authors declare that the research was conducted in the absence of any commercial or financial relationships that could be construed as a potential conflict of interest.

## Publisher’s Note

All claims expressed in this article are solely those of the authors and do not necessarily represent those of their affiliated organizations, or those of the publisher, the editors and the reviewers. Any product that may be evaluated in this article, or claim that may be made by its manufacturer, is not guaranteed or endorsed by the publisher.

## Footnotes

1
https://www.r-project.org/
