# Supplementary material for: Age Dependent Partitioning Patterns of Essential Nutrients Induced by Copper Feeding Status in Leaves and Stems of Poplar
Source: Front Plant Sci. 2022 Jul 5;13:930344. doi: 10.3389/fpls.2022.930344 (PMC9294533; doi:10.3389/fpls.2022.930344)
Supplement: Supplementary file 1 [file Data_Sheet_1.DOCX]

Supplementary Material

# Supplementary Figures and Tables

## Supplementary Figures


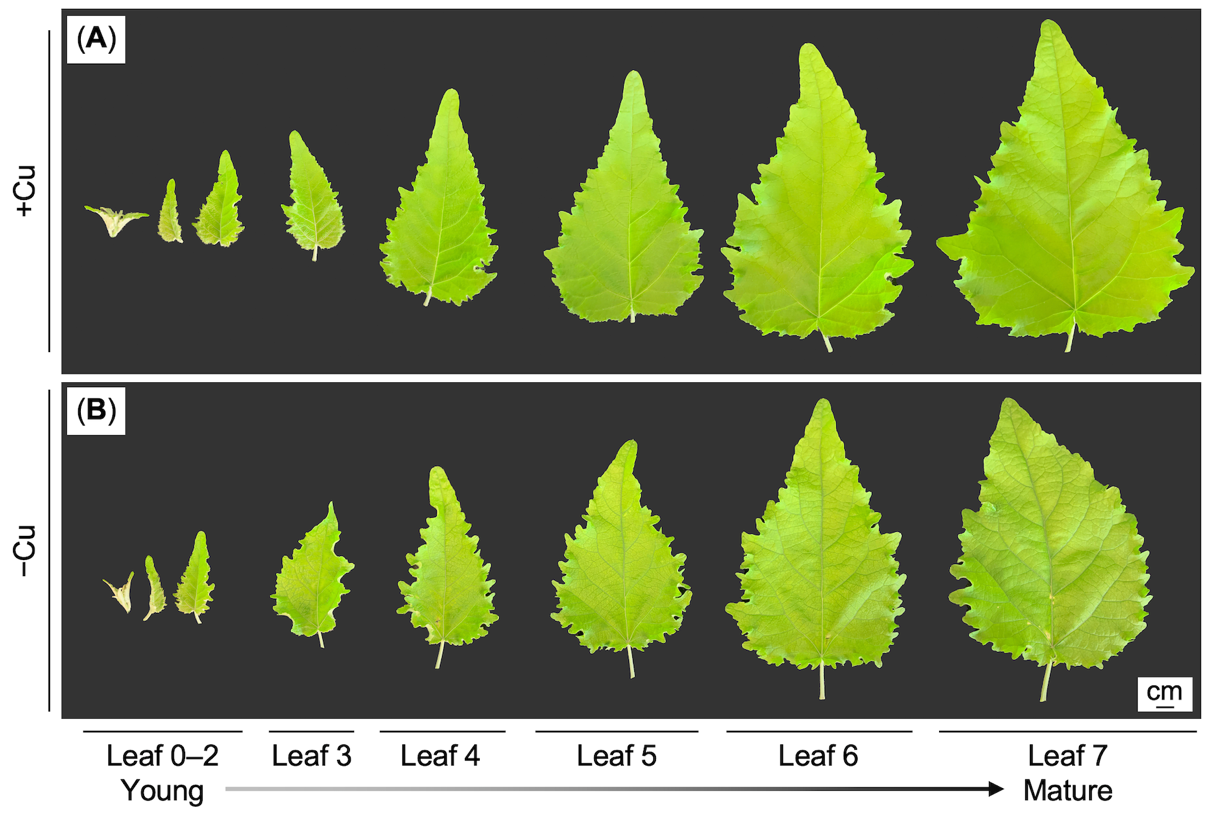


**Supplementary Figure 1.** Images of characterized leaves of hybrid white poplar (*P. tremula* × *P. alba*) grown in (**A**) +Cu and (**B**) −Cu conditions.


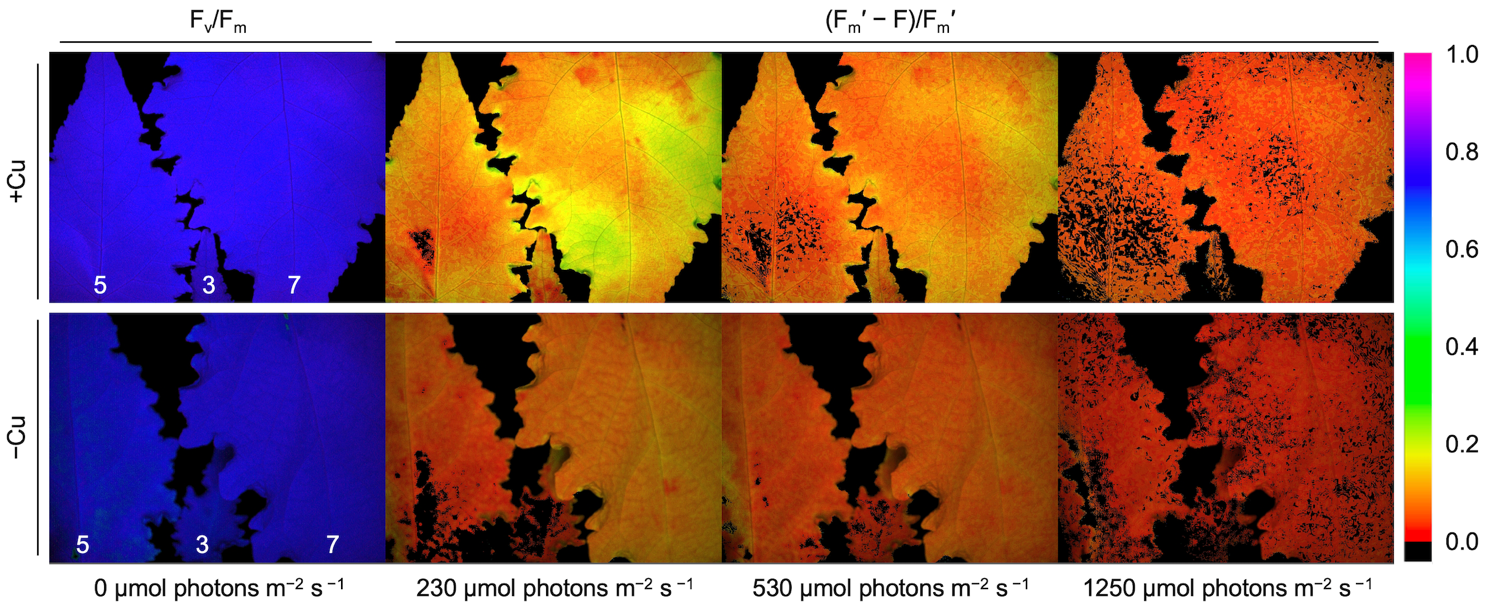


**Supplementary Figure 2.** Images with the response of photosystem II efficiency to increasing light intensity in Leaf 3, Leaf 5, and Leaf 7 (denoted by respective numbers in the first images) of hybrid white poplar (*P. tremula* × *P. alba*) grown in +Cu and −Cu conditions.
